# Niche Differentiation in the Composition, Predicted Function, and Co-occurrence Networks in Bacterial Communities Associated With Antarctic Vascular Plants

**DOI:** 10.3389/fmicb.2020.01036

**Published:** 2020-06-03

**Authors:** Qian Zhang, Jacquelinne J. Acuña, Nitza G. Inostroza, Paola Duran, María L. Mora, Michael J. Sadowsky, Milko A. Jorquera

**Affiliations:** ^1^The BioTechnology Institute, University of Minnesota, St Paul, MN, United States; ^2^Laboratorio de Ecología Microbiana Aplicada (EMALAB), Departamento de Ciencias Químicas y Recursos Naturales, Universidad de La Frontera, Temuco, Chile; ^3^Network for Extreme Environment Research (NEXER), Scientific and Technological Bioresource Nucleus (BIOREN), Universidad de La Frontera, Temuco, Chile; ^4^Department of Soil, Water, and Climate, and Department of Plant and Microbial Biology, University of Minnesota, St. Paul, MN, United States

**Keywords:** bacterial community, *Colobanthus quitensis*, *Deschampsia antarctica*, endosphere, phyllosphere, rhizosphere

## Abstract

Climate change directly affecting the Antarctic Peninsula has been reported to induce the successful colonization of ice-free lands by two Antarctic vascular plants (*Deschampsia antarctica* and *Colobanthus quitensis*). While studies have revealed the importance of microbiota for plant growth and stress tolerance in temperate climates, the role that plant-associated microbes play in the colonization of ice-free lands remains unknown. Consequently, we used high-throughput DNA sequence analyses to explore the composition, predicted functions, and interactive networks of plant-associated microbial communities among the rhizosphere, endosphere, and phyllosphere niches of *D. antarctica* and *C. quitensis*. Here we report a greater number of operational taxonomic units (OTUs), diversity, and richness in the microbial communities from the rhizosphere, relative to endosphere and phyllosphere. While taxonomic assignments showed greater relative abundances of *Proteobacteria, Bacteroidetes*, and *Actinobacteria* in plant niches, principal coordinate analysis revealed differences among the bacterial communities from the other compartments examined. More importantly, however, our results showed that most of OTUs were exclusively found in each plant niche. Major predicted functional groups of these microbiota were attributed to heterotrophy, aerobic heterotrophy, fermentation, and nitrate reduction, independent of plant niches or plant species. Co-occurrences network analyses identified 5 (e.g., *Microbacteriaceae, Pseudomonaceae, Lactobacillaceae*, and *Corynebacteriaceae*), 23 (e.g., *Chitinophagaceae* and *Sphingomonadaceae*) and 7 (e.g., *Rhodospirillaceae*) putative keystone taxa present in endosphere, phyllosphere, and rhizosphere, respectively. Our results revealed niche differentiation in Antarctic vascular plants, highlighting some putative microbial indicators and keystone taxa in each niche. However, more studies are required to determine the pivotal role that these microbes play in the successful colonization of ice-free lands by Antarctic plants.

## Introduction

Climate change has become of global concern over the last several decades. This is of particular importance to the polar regions of the world, such as the Antarctic Peninsula. Studies have reported that the Antarctic Peninsula has been subjected to recent warming and cooling events, suggesting the uncovering of new ice-free lands (Lee et al., 2017). This may subsequently lead to the greater availability of potentially new habitats for colonization by numerous organisms and a higher connectivity between habitats (Lee et al., 2017). Recent Antarctic cooling events have resulted in deleterious effect on lichens, which are a dominant vegetation type in the Antarctic peninsula, creating new opportunities for expansion by other vegetation species (Sancho et al., 2017). In this context, the expansion of Antarctic vascular plants has been attributed to their efficient nitrogen acquisition capacity, competing with both soil microorganisms and lichens (Hill et al., 2011). More recently, Royles et al. (2013) proposed that the increase in terrestrial plant growth rates and soil microbial activity are consistent with recent warming events on the Antarctic peninsula. Moreover, studies have also shown that warming due to global climate events have significantly influenced the abundance, composition, and activity of soil microorganisms from Antarctic environments (Yergeau et al., 2012).

Previous molecular studies have revealed that the rhizosphere (the soil portion influenced by roots) of Antarctic vascular plants, including Antarctic hair grass (*Deschampsia antarctica*) and Antarctic pearlwort (*Colobanthus quitensis*), can harbor a wide diversity of bacteria (Teixeira et al., 2010; Jorquera et al., 2016). Differences in bacterial community composition in the rhizospheres of *D. antarctica* and *C. quitensis* were observed by Teixeira et al. (2010) and members of *Firmicutes* were more abundant in the rhizosphere of *D. antarctica* compared to that of *C. quitensis*.

Results from several studies have established that bacteria are relevant for growth and tolerance of plants to harsh conditions in extreme environments. For example, a plant growth-promoting bacteria (PGPB) was isolated from the rhizosphere of *D. antarctica* showing the ability to promote the plant root development *in vitro* inoculation assay (Berríos et al., 2013). Similarly, the salt tolerance and ecophysiological performance of *D. antarctica* and *C. quitensis* was improved when plants were inoculated with Antarctic bacteria isolated from their rhizosphere (Gallardo-Cerda et al., 2018). Despite these advances, the contribution of microbiota from the endosphere (inner tissues of plants) and phyllosphere (the aerial part of plant leaves) to plant fitness have scarcely been considered, especially since these compartments are thought to be essential for plant success (Cid et al., 2017). In addition, new studies have revealed that the plant microbiome is structured and complex and interconnected by microbial networks (Turner et al., 2013; Vandenkoornhuyse et al., 2015; Banerjee et al., 2018). Moreover, these microbial networks harbor keystone taxa that act as drivers of the structure and functioning of microbiome and are likely essential for plant health and ecosystem functioning (van der Heijden and Hartmann, 2016; Banerjee et al., 2018). Evidence for such a scenario also comes from a recent study showing that plant and microbiome interactions are also complicated by plant-plant-microbe interactions (Molina-Montenegro et al., 2018).

As global climate issues become of even more concern, there is a need to better understand the diversity, functionality, and response of plant-associated microbes under climate change, as well as their relevance for Antarctic vascular plants expansion onto ice-free lands. Under this scenario, the main goals of the present study were to: (1) determine if the composition, predicted function, and networks of bacterial communities significantly differ among niches (rhizosphere, endosphere and phyllosphere) of the Antarctic vascular plants (*D. antarctica* and *C. quitensis*); and (2) at the same time to identify putative microbial indicators and keystone taxa in each niche which may give cues on microbiota playing pivotal roles in the growth and/or colonization of ice-free lands by these plants.

## Materials and Methods

### Sampling

Plant specimens and their respective rhizosphere soils were collected during Antarctic Scientific Expedition no. 53 (ECA53; February 2017) to the South Shetland Islands of Antarctica, organized by Chilean Antarctic Institute (INACH). The plant specimens were taken from mantles of *D. antarctica* and *C. quitensis* located at the following coordinates: 62°59′53″S, 60°35′17″W and 62°24′7″S, 58°18′29″W, respectively. The plant specimens were randomly taken in a 10 m transect by using a clean spade to remove intact roots from soil. Collected plants and soils were placed within plastic bags, stored at 4°C, and transported on ice to the Applied Microbial Ecology Laboratory (EMALAB) at La Frontera University for microbiological analyses.

Endosphere samples from four plants of each species were processed as described by Barra et al. (2016). Plant tissues (roots and leaves) were washed and surface sterilized by repeated immersion in 70% (v/v) ethanol for 3 min, followed by 2.5% (v/v) sodium hypochlorite (NaOCl) for 5 min, and exhaustive rinsing with sterile distilled water (SDW). Portions of tissues (1–2 g) were aseptically cut, frozen in liquid nitrogen, macerated and homogenized with a mortar and pestle, and stored at −80°C until DNA extraction. In parallel, quadruplicate phyllosphere leaf samples were processed as described by Cid et al. (2017). Briefly, 1 g portions of leaves were cut (aerial parts), gently washed, and vortexed for 10 min in 10 ml sterile saline solution (0.85% NaCl). Leaves were removed, and the recovered liquid was centrifuged at 15,700 × g for 10 min to collect detached bacterial cells. Bacterial cells were suspended in 50 μl of SDW, and this suspension was subsequently frozen in liquid nitrogen and thawed at room temperature three times. Samples were centrifuged at 15,700 × g for 40 min, and the supernatant (~40 μl) was used as template DNA in PCR reactions. Rhizosphere soil samples from each plant specimen were processed, in quadruplicate, as described by Lagos et al. (2014). Briefly, soil aggregates were detached from roots by vigorous vortexing and collected in sterile polypropylene microtubes. Rhizosphere soils were gently mixed, and 1–2 g subsamples were stored at −80°C, and later subjected to DNA extraction.

The physicochemical properties of the rhizosphere soils were also determined as follow. The pH was measured in 1:2.5 soil/deionized water suspensions. Available phosphorus (P_Olsen_) was extracted using 0.5 M Na-bicarbonate method and analyzed using the molybdate-blue method (Murphy and Riley, 1962). Organic matter contents were estimated by wet digestion (Walkley and Black, 1934). Exchangeable cations (K^+^, Ca^2+^, Mg^2+^, and Na^+^) were extracted with 1M CH_3_COONH_4_ at pH 7.0 and analyzed using flame atomic adsorption spectrophotometry (FAAS) (Warncke and Brown, 1998). Exchangeable aluminum (Al^3+^) was extracted with 1M KCl and analyzed by FAAS (Bertsch and Bloom, 1996).

### DNA Extraction

DNA from the endosphere and phyllosphere samples was extracted by using Quick–DNA^TM^ Plant/seed Miniprep kits (Zymo Research, CA, USA). DNA from rhizosphere soil samples was extracted with PowerSoil® DNA isolation kit (Qiagen, MO BIO Laboratories, CA, USA), both kits were used according to manufacturer instructions.

### High–Throughput DNA Sequencing

The distribution and relative abundances of endophytic bacteria in root endospheres, leaf phyllospheres, and rhizosphere soils, was assessed by high throughput DNA sequencing (HTS) analyses as follow. The V4 hypervariable region of the 16S rRNA was amplified, for bacteria and archaea, by using primer set 515F (5′- GTG CCA GCM GCC GCG GTA A−3′) and 806R (5′- GGA CTA CHV GGG TWT CTA AT−3′). Sequencing was done by the University of Minnesota Genomics Center (UMGC, Minneapolis, MN, USA) using barcoded primers and the dual indexing method (Gohl et al., 2016). Amplicons were gel purified, pooled, and paired–end sequenced at a read length of 300 nt on the Illumina MiSeq platform (Illumina, Inc., San Diego, CA, USA).

### Bioinformatics and Statistical Analysis

Mothur ver. 1.34.0 was used for most sequence analyses (Schloss et al., 2009). In brief, after trimming low-quality regions at the ends of reads, the paried-end sequencing reads were merged by Fastq-join software (Aronesty, 2013), maintaining an average quality score >33. Primer sequences were removed from reads and high quality sequencing reads were aligned on the basis of the Greengenes ver.13.8 (McDonald et al., 2012). The UCHIME software package was used to identify and remove probable chimeric sequences (Edgar et al., 2011). Non-microbiota (e.g., chloroplast and mitochondria) sequence reads were removed via QIIME (Caporaso et al., 2010), and data was rarefied to 14,000 sequence reads per sample set prior to statistical analysis. Raw sequencing data were deposited in the Sequence Read Archive (SRA) of NCBI under Accession Number PRJNA509213. For statistical analysis, the mothur program was also used to calculate alpha diversity indices, including Good's coverage, the Shannon index, and the Abundance-based Coverage Estimate (ACE). Principal coordinate analysis (PCoA) was used to ordinate the samples. Differences in beta diversity among the community were evaluated by analysis of similarity (ANOSIM) using Bray-Curtis dissimilarity matrices (Bray and Curtis, 1957; Clarke, 1993). Molecular variance (AMOVA) was used to measure differences in sample clustering (Excoffier et al., 1992). The VennDiagram package in R (https://www.r-project.org/) was used to identify shared OTUs of bacterial communities between plant niches (Chen and Boutros, 2011). Putative indicator OTUs in Antarctic vascular plants that were in association with the differentiation of plant niches were identified on the basis of the *multipatt* function using the *indicspecies* package in R (de Caceres and Legendre, 2009). The associations were further considered to be significant by using a false discovery rate (*q* < 0.1) (Strimmer, 2008). Visualization of the putative association of indicators with plant niches in the Antarctic plants was produced by using *gplots* package in R package (heatmap) and by iTOL (tree) (Letunic and Bork, 2016). FAPROTAX was used to predict potential functions among members of the microbial community in the different niches (rhizosphere, endospheres, and phyllosphere). Potential functions were determined via the default settings on the basis of taxonomic information of microbiota in Antarctic vascular plant (Louca et al., 2016).

### Network Analysis of Bacterial Community in Various Niches of Antarctic Vascular Plants

Rare microorganisms were defined as those that were not found above 0.01% relative abundance in the rhizosphere, endosphere and phyllosphere samples from *D. antarctica* and *C. quitensis*. The co-occurrence network was constructed as described by Ma et al. (2016). Briefly, a Spearman correlation matrix was used to generate the co-occurrence network via the WGCNA package. The nodes indicate the OTUs, while the edges, which are connecting the nodes, represent correlations between OTUs. Prior to network construction, random matrix theory (RMT) was performed to identify the appropriate similarity of 0.82 as the threshold (Luo et al., 2006), and the *P-*values of correlations were defined by using the Benjamini and Hochberg false discovery rate (FDR) of < 0.05 (Benjamini et al., 2006). The *igraph* package was used to measure the network properties (Csardi and Nepusz, 2006), while Gephi was further used to achieve the network image and calculations of closeness centrality and betweenness centrality for each node (Bastian et al., 2009). In addition, the occurrence of putative keystone taxa, which play pivotal roles in the structure and functioning of the microbial community (Banerjee et al., 2018), was determined in each niche as follows (Berry and Widder, 2014): For the endosphere network, OTUs with degree > 8, closeness centrality > 0.18, and betweenness centrality < 0.05 were used to identify putative keystone taxa. For the phyllosphere network, OTUs with > 5 degrees, closeness centrality > 0.15, and betweenness centrality < 0.05 were selected as putative keystone taxa. For the rhizosphere network, OTUs with > 7 degrees, closeness centrality > 0.17, and betweenness centrality < 11 were chosen as putative keystone taxa.

## Results

### Physicochemical Properties of Rhizosphere Soils

The physicochemical properties of triplicate rhizosphere soils are summarized in Table 1. These analyses revealed differences between rhizosphere soils from both Antarctic plants. Rhizosphere soils from *D. antarctica* showed higher contents of available P, K, and organic matter compared with that from *C. quitensis*. In contrast, rhizosphere soils from *C. quitensis* had a greater higher cation exchange capacity (CEC) and Al saturation compared with those from *D. antarctica*. Despite these differences, both plant species had rhizosphere soils with similar pH_H2O_ values, 6.1 and 6.3 for *D. antarctica* and *C. quitensis*, respectively.

**Table 1:** Physicochemical properties of rhizosphere soil samples from the Antarctic vascular plants used in this study.

	***Deschampsia antarctica***	***Colobanthus quitensis***
P_Olsen_ (mg kg^−1^)	85 ± 41.6^†^	25 ± 4.2
K (mg kg-^1^)	487.3 ± 85.9	277.7 ± 40.8
pH_H2O_	6.1 ± 0.2	6.3 ± 0.2
Organic matter (g kg^−1^)	9.2 ± 4	1.9 ± 0.7
K (cmol_(+)_ kg^−1^)	1.2 ± 0.2	0.7 ± 0.1
Na (cmol_(+)_ kg^−1^)	5.3 ± 1.6	1.6 ± 0.2
Ca (cmol_(+)_ kg^−1^)	10.3 ± 2.8	14.4 ± 2.8
Mg (cmol_(+)_ kg^−1^)	6 ± 0.9	7.7 ± 0.9
Al (cmol_(+)_ kg^−1^)	0.013 ± 0.03	0.057 ± 0.024
Al saturation (%)^‡^	0.063 ± 0.012	0.267 ± 0.136
CEC (cmol_(+)_ kg^−1^)	22.8 ± 3.9	24.5 ± 3.3
Σ bases (cmol_(+)_ kg^−1^)	22.8 ± 3.9	24.5 ± 3.4

† *The values represent means ± standard errors from n = 3*.

‡ *Calculated as (Al× 100) / CEC, where CEC=cation exchange capacity = Σ (K, Ca, Mg, Na, and Al)*.

### Coverage and Alpha Diversity of Bacterial Community

Sequencing resulted in an estimated 98 to 99% coverage of OTUs in the endosphere and phyllosphere samples from both Antarctic plants. Substantial, but significantly (Tukey's *post–hoc* test, *p* < 0.05) lower coverage (95 to 96%) was observed in the rhizosphere samples relative to those from the other plant niches (Table 2). Similarly, a significantly (*p* < 0.05) greater number of OTUs (define at a 97% similarity) was observed in rhizosphere samples (1,551 and 1,628 for *D. antarctica* and *C. quitensis*, respectively), compared with those found in other plant niches. In this sense, the numbers of OTUs observed in the endosphere and phyllospheres samples were lower in *D. antarctica* (434 and 522 OTUs, respectively) compared to those in *C. quitensis* (662 and 666 OTUs, respectively). Bacterial alpha diversity, revealed by the Shannon index, was significantly (*p* < 0.05) greater in rhizosphere samples (6.2 for both plant species) compared with endospheres (3.9 and 4.9 for *D. Antarctica* and *C. quitensis*, respectively) and phyllospheres (3.7 and 4.4 for *D. Antarctica* and *C. quitensis*, respectively). In addition, significantly lower ACE values (*p* < 0.05) were observed in the endosphere (525 and 884 for *D. Antarctica* and *C. quitensis*, respectively) and phyllosphere (865 and 1,312 for *D. Antarctica* and *C. quitensis*, respectively) samples compared with those in the rhizospheres (2,093 and 2,237 for *D. Antarctica* and *C. quitensis*, respectively).

**Table 2:** Coverage and alpha diversity (mean ± standard deviation) among bacterial communities by endosphere, phyllosphere, and rhizosphere in two Antarctic vascular plants, based on high–throughput DNA sequencing data in each plant species (*n* = 4).

**Plant**	**Niche**	**Coverage (%)**	**S_obs_^†^**	**Shannon index**	**ACE^‡^**
*Deschampsia antarctica*	Endosphere	99.25 ± 0.34^A^^*^	434 ± 177^A^	3.93 ± 1.06^A^	525 ± 206^A^
	Phyllosphere	98.58 ± 1.41^A^	522 ± 576^A^	3.74 ± 1.49^A^	865 ± 754^A^
	Rhizosphere	96.33 ± 0.46^B^	1551 ± 66^B^	6.23 ± 0.15^B^	2093 ± 186^B^
*Colobanthus quitensis*	Endosphere	98.59 ± 1.12^A^	662 ± 458^A^	4.34 ± 1.29^A^	884 ± 615^A^
	Phyllosphere	98.07 ± 0.86^A^	666 ± 296^A^	4.36 ± 0.66^A^	1312 ± 544^A^
	Rhizosphere	95.98 ± 0.51^B^	1628 ± 188^B^	6.25 ± 0.21^B^	2237 ± 260^B^

† *S_obs_: number of OTUs observed at 97% similarity*.

‡ *ACE: abundance-based coverage estimate*.

* *Sample groups sharing the same letter in each niche did not vary significantly (P ≤ 0.05) by ANOVA followed by Tukey's post-hoc test*.

### Taxonomy Assignments of Bacterial Community

**Figure 1:**
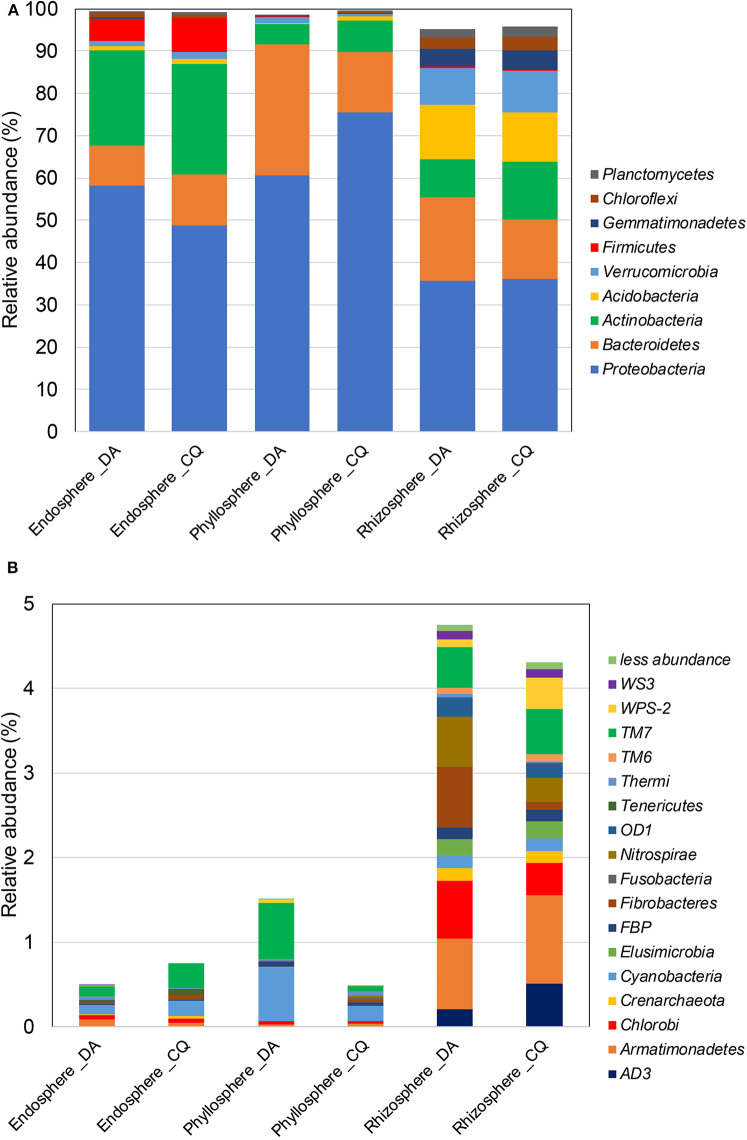
Mean relative abundances of major **(A)** and minor **(B)** phylum-level taxa of bacterial communities in the endosphere, phyllosphere, and rhizosphere of the Antarctic vascular plants *Deschampsia antarctica* (DA) and *Colobanthus quitensis* (CQ).

Assignment of taxonomic affiliation to members of bacterial communities indicated that members of the *phylum Proteobacteria* were relatively abundant in both Antarctic plants, with values of 48.8 to 58.9, 60.6 to 75.4, and 35.7 to 36.1% for endosphere, phyllosphere, and rhizosphere samples, respectively (Figure 1). While the endosphere communities in both plant species were also colonized by relatively large numbers of *Actinobacteria* (22.6 to 26.1%), *Bacteroidetes* (9.3 to 12%), and *Firmicutes* (5.3 to 7.9%) phyla, the phyllosphere was co-dominated by members of the phyla *Bacteroidetes* (14.3 to 30.9%) and *Actinobacteria* (4.8 to 7.5%). In contrast, the rhizosphere was also co-dominated by members of the phyla *Bacteroidetes* (14 to 19.7%), *Acidobacteria* (11.7 to 12.8%), *Actinobacteria* (9.2 to 13.7%), and *Verrucomicrobia* (8.7 to 9.9%). With respect to minor taxa, those present at < 10% of communities, a higher presence of bacterial groups was observed in rhizosphere samples compared with those in other niches, and were mainly attributed to members of the phyla *Armatimonadetes* (0.8 to 1%), *Chlorobi* (0.4 to 0.7%), *Saccharibacteria* (formerly TM7) (0.5%), and *Nitrospirae* (0.3 to 0.6%) in the rhizosphere of both plant species.

**Figure 2:**
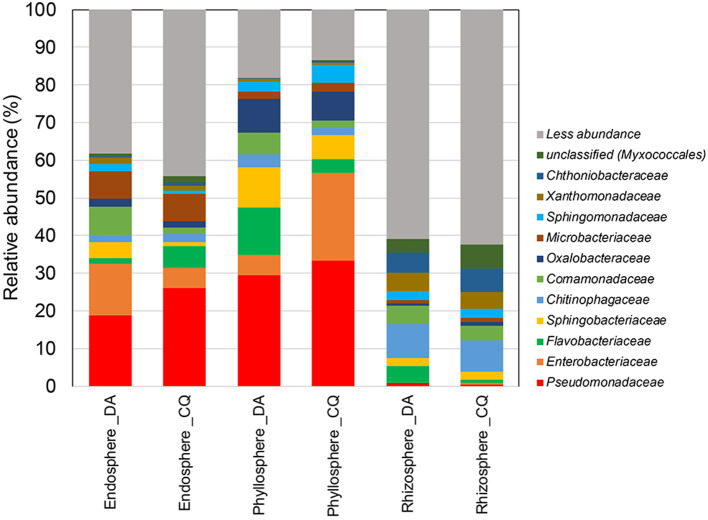
Mean relative abundances of family-level taxa of bacterial communities in the endosphere, phyllosphere, and rhizosphere of the Antarctic vascular plants *Deschampsia antarctica* (DA) and *Colobanthus quitensis* (CQ).

At the family level, a greater relative abundance of taxa in the endosphere were attributed to *Pseudomonadaceae* (18.7 to 26.2%), followed by *Enterobacteriaceae* (5.3 to 13.8%), and *Microbacteriaceae* (7.2 to 7.4%) (Figure 2). A greater diversity of families was observed in the phyllosphere samples, with higher relative abundances of *Pseudomonadaceae* (25.5 to 33.3%) followed by *Enterobacteriaceae* (5.3 to 23.4%), *Sphingobacteriaceae* (6.5 to 10.7%), *Oxalobacteriacea* (7.7 to 9%), and *Flavobacteriaceae* (5.6 to 12.7%) families. Interestingly, *Pseudomonadaceae* were found as an abundant group in the endospheres and phyllospheres, but not in the rhizospheres. In contrast, the rhizosphere samples were dominated by members of the *Chitinophagaceae* (8.3 to 8.9%) followed by *Chthoniobacteraceae* (5.3 to 6.3%), *Xanthomonadaceae* (4.4 to 4.9%), and *Comamonadaceae* (3.8 to 4.8%) families.

### Unique Microbial Communities Are Revealed by Beta Diversity Analyses

**Figure 3:**
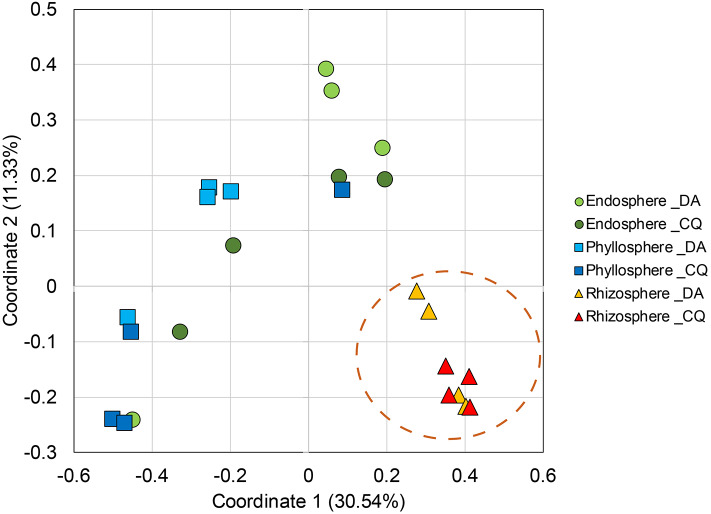
Principal coordinate analysis (PCoA) of Bray-Curtis dissimilarity matrices of bacterial communities in the endosphere, phyllosphere, and rhizosphere of the Antarctic vascular plants *Deschampsia antarctica* (DA) and *Colobanthus quitensis* (CQ) (*r*^2^ = 0.57). Pairwise comparison (Bonferroni) of bacterial communities between three plant compartments were performed by ANOSIM.

**Figure 4:**
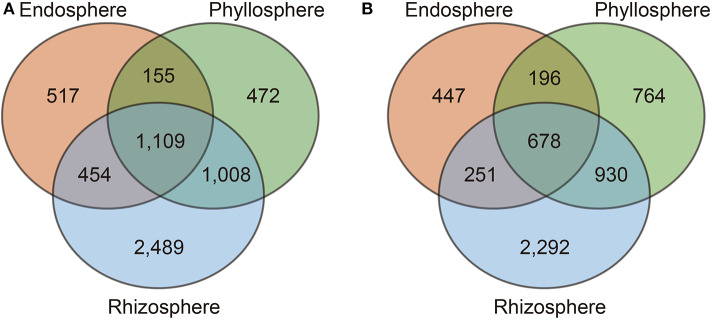
Shared operational taxonomic units (OTUs) among bacterial communities present in the endosphere, phyllosphere, and rhizosphere of the Antarctic vascular plants *Deschampsia antarctica* **(A)** and *Colobanthus quitensis* **(B)**.

PCoA analyses showed a clear separation between microbiota in the rhizosphere and other plant niches in both Antarctic plants (Figure 3), but these differences were not seen between plant species. Our analysis also revealed that 24.2% (1,109 of 4,587) and 16.2% (678 of 4,181) of OTUs were shared between the three plant niches of *D. antarctica* and *C. quitensis*, respectively (Figure 4). In contrast, 75.8% (3,478 of 4,587) and 83.2% (3,503 of 4,181) of the OTUs were not shared and they were exclusively found in the individual plant niches of *D. antarctica* and *C. quitensis*, respectively. The greatest number of unique, not shared, sequences were found in rhizosphere samples, with 2,489 and 2,293 OTUs for *D. antarctica* and *C. quitensis*, respectively. The detailed distribution of shared and unique OTUs among bacterial communities in the plant niches are also shown in Table S1.

### Predicted Functions of Bacterial Community Members

**Figure 5:**
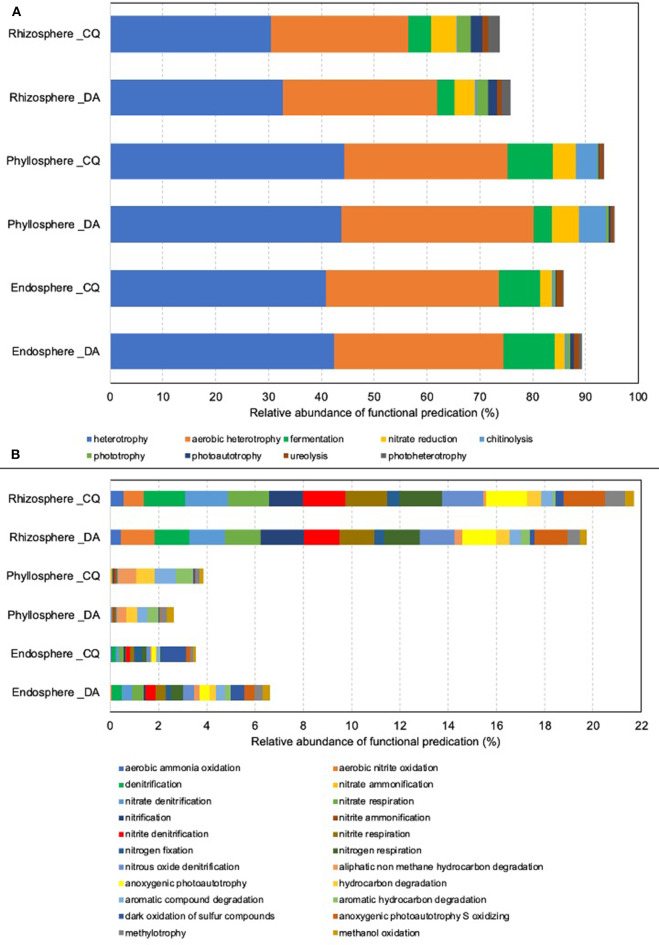
Mean relative abundances of microbial functional groups in the rhizosphere, endosphere, and phyllosphere of *Deschampsia Antarctica and Colobanthus quitensis*. **(A)** Major functional groups and **(B)** minor functional groups.

Presumptive microbial functional groups in each plant niches are shown in Figure 5. Independent of niches and species, the major functions were attributed to heterotrophy (30.5 to 44.3%) and aerobic heterotrophy (25.8 to 36.3%) (Figure 5A). Fermentation (3.4 to 9.7%) and nitrate reduction functions (1.8 to 5.2%) were also assigned in all niches. When minor functional groups were analyzed, a greater abundance of functional assignments were observed in rhizospheres, compared to those from the endosphere and phyllospheres (Figure 5B). A greater abundance of functions related to nitrogen cycling was observed in the rhizosphere of *D. antarctica* and *C. quitensis*. In contrast, in the phyllosphere samples the functions were mainly attributed to degradation of aliphatic and aromatic hydrocarbons. Lastly, samples from the endosphere also showed functions related to nitrogen cycling and hydrocarbon degradation, and dark oxidation of sulfur compounds.

### Microbial Indicators of Niches in Antarctic Plants

**Figure 6:**
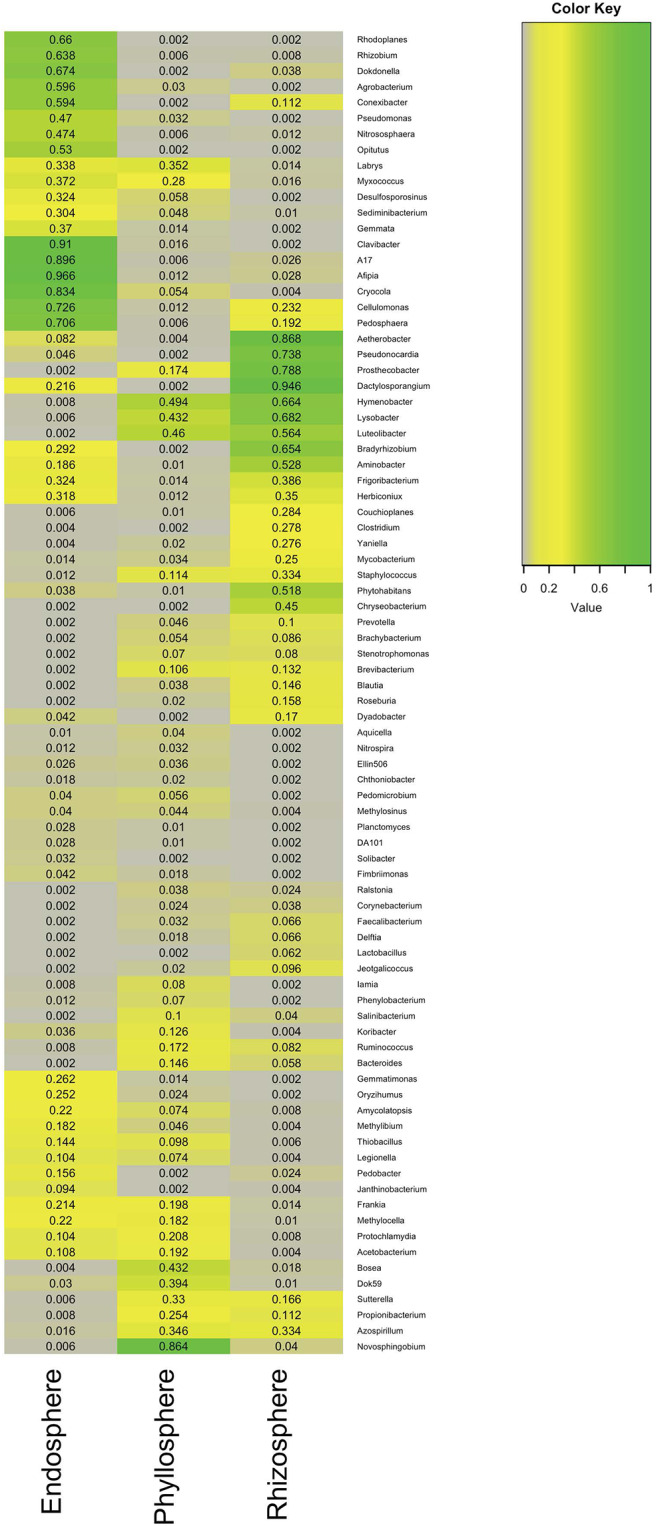
Indicator heatmap showing the taxonomy and taxon-treatment-association strength of 84 microbial genera significantly (*q* < 0.1) associated with different compartments within Antarctic plants. The values represent the association strength.

Indicator analyses, based on taxonomic assignments from the genus to phylum levels, was used to investigate the association between taxon abundance and plant niches. Detailed information on the average abundance of each bacterium in each niche, their maximum association, the significance of associations (*p*-value), and the false discovery rate correction value (*q*) can be found in Figure 6 and Figure S1 and Table S2. Overall, our analyses identified 256 OTUs that were significantly associated with various plant niches. Of these, however, only 84 taxa could be classified to the genus-level. While these 84 taxa were distributed among 12 phyla, most belonged to the *Proteobacteria, Actinobacteria*, and *Firmicutes*. Notably, *Pseudomonas*, which had the greatest abundance among the three plant niches, could be used as putative indicator taxa in the endosphere, where it was significantly associated with this niche. Moreover, *Clavibacter* was also greatly associated (R=0.91) with the endosphere. In contrast, *Novosphingobium* was the best putative indicator bacterium representing the phyllospheres, although it had the highest association (R=0.86) among the three niches. In contrast, *Dactylosporangium* (R=0.95) and *Bradyrhizobium* (R=0.65) were the best putative indicators of the rhizosphere niche in Antarctic plants (R=0.95), and.

### Niche-Specific Co-occurrence Networks in Antarctic Plants

**Figure 7:**
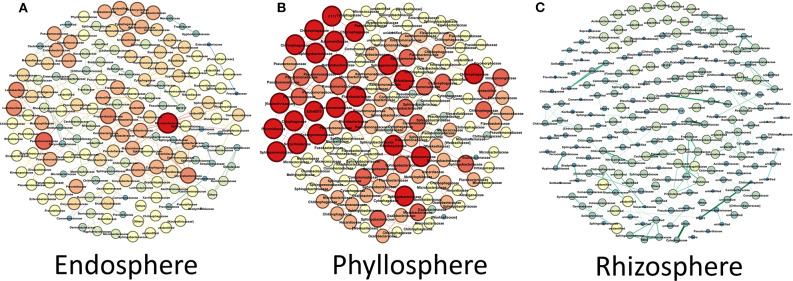
Antarctic plant niche-specific co-occurrence networks among microorganisms in the **(A)** endosphere, **(B)** phyllosphere, and **(C)** rhizosphere. The size of each node (OTU) is the proportional to the number of connections.

Due to the differences in microbial community structure and microbiota composition across the three plant niches, we further investigated the bacterial network and putative keystone taxa for each niche separately (Figure 7 and Tables S3–S5). Network analysis of the endosphere included 842 nodes (e.g., OTUs) and 1,062 edges, indicative of the association between OTUs. Results in Figure 7A show five putative keystone taxa in the endosphere network: *Microbacteriaceae* (0.05%), *Pseudomonadaceae* (0.6%), two *Lactobacillaceae* (0.14% and 0.06%), and *Corynebacteriaceae* (0.02%). Most notably, the family *Microbacteriaceae* was the most critical keystone taxon, bridging the maximum number of nodes and associations in the endosphere network. The family *Pseudomonadaceae*, which had greater relative abundance among these five putative keystone taxa identified, was coordinated with *Pseudomonas* as an putative indicator bacterium at the genus-level in the endosphere (Figures 6, 7A and Figure S1). In contrast, network analysis of the phyllosphere identified only 567 nodes and 386 edges. Impressively, although the phyllosphere network had fewer nodes and edges, 23 putative keystone taxa were identified (Figure 7B). The majority of the associations in the phyllosphere were from the families *Chitinophagaceae* (4 out of 23) and *Sphigomonadaceae* (3 out of 23). In the case of the phyllosphere, *Sphigomonadaceae*, in association with *Novosphingobium*, at the genus-level, was the best putative indicator in the phyllosphere samples (*R* = 0.86). In contrast, and perhaps expected due to its high diversity, the rhizosphere network consisted of 1,392 nodes and 2,682 edges, by far the largest of the three compartmental niches. Despite its large size, however, this highly complex rhizosphere network only had 7 putative keystone taxa (Figure 7C). Moreover, among these seven putative keystone taxa, only *Rhodospirillaceae* was identified at the family-level.

## Discussion

During the last 30 years, climate change has influenced the distribution and abundance of species worldwide and is attributed to be a major cause of the acceleration of world-wide species extinction (Urban, 2015). The polar regions are not an exception, and climate change has also affected the ecology of plants and animals in Arctic and Antarctic ecosystems. In this sense, the successful expansion of vascular plants on the Antarctic peninsula has been attributed to the impact of climate change (Lee et al., 2017). Similarly, it has been reported that invasion of generalist microbes from warmer latitudes will replace many local specialist microbes, and this along with the retraction and losses of ice, will further reduce opportunities for niche specialization (Vincent, 2010). Thus, given the importance of microbes on the growth, fitness and productivity of plants (Turner et al., 2013; Vandenkoornhuyse et al., 2015), additional studies are needed to better understand the impacts of climate change on the colonization of ice-free lands by Antarctic vascular plants and the extinction risks to species in polar regions.

In this study, we examined the diversity and richness of plant-associated microbiota in three plant compartments (niches) of Antarctic vascular plants. Differences in microbial community structure were found in the rhizosphere compared with the endosphere and phyllosphere. The rhizosphere is considered as the main hotspot for microbial colonization and activity in soils, harboring a great abundance and diversity of bacteria compared with other plant and soil niches (Prashar et al., 2014). In contrast, the endosphere is considered as a restricted niche where colonization by endophytes depends of diverse variables associated with the degree of intimacy between endophytic bacteria (e.g., opportunistic or facultative) and the host plant. Another recent study also pointed out that penetration route (e.g., root hairs, stomata, flower, etc.), plant genotype, and strain type, also have a large impact on colonization (Hardoim et al., 2015). Similarly, the phyllosphere has been categorized as a hostile environment to bacteria and is governed by diverse abiotic factors (such as ultraviolet radiation, temperature, desiccation, etc.) that can change within few minutes, hours, days, or even seasons (Yang et al., 2001; Lindow and Brandl, 2003). Therefore, only adapted, non-fastidious bacterial populations can survive and/or proliferate in the endospheres and phyllospheres of Antarctic vascular plants.

Independent of plant species or niche studied, our Illumina-based sequence analyses revealed the dominance of members of the phyla *Proteobacteria* in all studied bacterial communities. Members of the *Proteobacteria* have been found to be dominant in plant niches, including the endosphere (Hardoim et al., 2015; Proença et al., 2017; Yang et al., 2017), phyllosphere (Whipps et al., 2008; Tian et al., 2017) and rhizosphere (Wang et al., 2018; Lei et al., 2019). Coincidently, our study also showed that members of phyla *Actinobacteria* and *Bacteroidetes* were dominant in both Antarctic plants. Several other studies have shown that the *Actinobacteria* and *Bacteroidetes* are the dominant bacterial groups in the plant microbiome (Turner et al., 2013; Vandenkoornhuyse et al., 2015; Venkatachalam et al., 2016). This association has also been noted in relation to the rhizosphere and phyllosphere of Antarctic vascular plants (Teixeira et al., 2010; Jorquera et al., 2016; Cid et al., 2017; Molina-Montenegro et al., 2018).

Interestingly, our study also revealed that the majority of OTUs observed were not shared, particularly those found in the rhizosphere samples. These findings are consistent with studies showing niche differentiation in plants (Coleman-Derr et al., 2016; Beckers et al., 2017; Cheng et al., 2018; Rilling et al., 2018). Similarly, one of our recent studies revealed that most of OTUs were not shared between endospheres (leaves and roots) of indigenous plants, suggesting the effect of the plant genotype (species) on the bacterial endophyte communities in Chilean extreme environments (Zhang et al., 2019). This niche differentiation might be influenced by a combination of different factors, including the chemical properties of rhizosphere soil as observed in our analysis (Table 1), and as suggested by Rilling et al. (2018).

With respect to the predicted functions of bacterial communities, our study showed major assignments to heterotrophy, aerobic heterotrophy, fermentation, and nitrate reduction. In a minor degree, functions were also found to be related to nitrogen cycling and hydrocarbon degradation were found. Functional studies in bacterial communities associated with Antarctic vascular plants are very limited. A recent metagenomic study observed revealed a higher diversity of functional genes and abundance of stress tolerance genes in the rhizosphere of *D. antarctica* plus *C. quitensis* than *C. quitensis* (Molina-Montenegro et al., 2018). In addition, the sequencing of genome from culturable bacteria isolated from *D. antarctica* phyllosphere, showed genes associated with nutrient uptake, bioactive metabolites, and antimicrobial compounds (Cid et al., 2018). Similar to our study, *Pseudomonas* are commonly found in the rhizospheres and phyllospheres of Antarctic vascular plants (Teixeira et al., 2010; Peixoto et al., 2016; Cid et al., 2017). *Pseudomonas* is recognized as a metabolically versatile bacterial group that exhibits a wide battery of activities such as nutrient cycling, degradation of organic compounds, among others (Timmis, 2002; Loeschcke and Thies, 2015). However, it is necessary to mention that our results are predictive, based on 16S rRNA gene sequences, and the functionality of microbiota in each plant species must be determined by experimental setting *in vitro* and *in situ*.

Microbial indicator analyses allowed identification of taxa mainly clustered into the phyla *Proteobacteria, Actinobacteria*, and *Firmicutes*, independent of niche and plant species. Members of *Proteobacteria, Actinobacteria*, and *Firmicutes* phyla are frequently reported as the dominant bacterial taxa in soil and plant microbiome in Arctic and Antarctic environments (Teixeira et al., 2010; Jorquera et al., 2016; Peixoto et al., 2016; Poosakkannu et al., 2017; Molina-Montenegro et al., 2018). In relation to three niche co-occurrence networks, our results showed significant difference among the plant niches investigated, where 5 (*Microbacteriaceae, Pseudomonaceae, Lactobacillaceae*, and *Corynebacteriaceae*), 23 (*Chitinophagaceae* and *Sphingomonadaceae*), and 7 (*Rhodospirillaceae*) major putative keystone taxa at family-level were observed in endosphere, phyllosphere and rhizosphere, respectively. *Rhizobium* spp. have been proposed as keystone taxa *in planta*, whereas *Gemmatimonas* and *Acidobacteria* have been proposed in soil (Banerjee et al., 2018). In Chilean extreme ecosystems, we recently reported to *Bacillaceae* and *Enterobacteriacea* as keystone taxa in endophytic bacterial communities associated with plants (Zhang et al., 2019).

To our knowledge, the occurrence of keystone taxa in Antarctic plants has not been reported thus far, so comparison to other studies are difficult. Moreover, because there are scarce studies simultaneously analyzing different niches in plants, comparisons of the putative microbial indicators or keystone taxa proposed here with those in related plant species grown in other continents is difficult. That said, however, the diversity of microbiomes across plant niches (leaves, stems, roots and soils) for *Populus* trees has been reported (Cregger et al., 2018). Even without network analyses, some studies have shown plant host-induced microbial populations changes. For example, studies on rhizobacterial communities associated with *Deschampsia caespitosa*, a metal-tolerant plants in European heavy metal polluted soils, revealed that the *Cytophagaceae* family is a specie-specific dominant group, and that there are distinctive profiles of microbial traits that are influence by soil properties and plant genotype (Cavalca et al., 2015; Borymski et al., 2018).

This study represents our first directed approach to examine the influence of environmental and biological factors (e.g., season, weather, and plant development) on niche differentiation of microbial communities in Antarctic vascular plants. This analysis suggests the presence of putative niche-specific microbial indicators and major keystone taxa. However, further investigations, including longitudinal gradient samplings, are required to demonstrate if specific bacterial communities (or specific group) are pivotal to the successful colonization (or expansion) of ice-free lands by plants in the Antarctic.

## Data Availability Statement

The datasets generated for this study can be found in the Raw sequencing data were deposited in the Sequence Read Archive (SRA) of NCBI under Accession Number PRJNA509213.

## Author Contributions

QZ, JA, NI, MS, and MJ designed the research and performed laboratory work and data analysis. QZ, MS, and MJ wrote the manuscript and designed tables and figures. JA, PD, MS, and MM made critical revisions of the main manuscript. All authors revised the manuscript and approved the final version.

## Conflict of Interest

The authors declare that the research was conducted in the absence of any commercial or financial relationships that could be construed as a potential conflict of interest.
