# Supplementary material for: Niche Differentiation in the Composition, Predicted Function, and Co-occurrence Networks in Bacterial Communities Associated With Antarctic Vascular Plants
Source: Front Microbiol. 2020 Jun 3;11:1036. doi: 10.3389/fmicb.2020.01036 (PMC7285837; doi:10.3389/fmicb.2020.01036)
Supplement: Supplementary file 1 [file Data_Sheet_1.zip › Data Sheet 1.PDF]

Supplementary Table S1. Distribution of shared and unique operational taxonomic units (OTUs) among bacterial communities in the endosphere (E), phyllosphere (P) and rhizosphere (R) of two Antarctic vascular plants, based on high–throughput DNA sequencing data in each plant species (*n*=4).

	Deschampsia antarctica				***Colobanthus quitensis***			
	**Shared OTUs**	**Unique OTUs**			**Shared OTUs**	**Unique OTUs**		
**Taxa (phylum)**		**E**	**P**	**R**		**E**	**R**	**R**
p_unclassified				3				1
Acidobacteria	64	2	9	374	23	2	37	359
Actinobacteria	196	88	82	211	157	48	138	197
AD3				5				7
Armatimonadetes	3	1	2	40	3		3	49
Bacteroidetes	198	15	87	294	97	66	147	223
BHI80-139				2				1
BRC1				3			1	2
Chlamydiae				2			1	5
Chlorobi	2			7	15	1	1	4
Chloroflexi	28	2	3	48			12	55
Crenarchaeota	2			1		1		4
Cyanobacteria	4	3	2	16	5		6	19
Elusimicrobia			1	15				17

Euryarchaeota				7				1
FBP	4		3	1	2		5	9
Fibrobacteres	1		2	73	1			2
Firmicutes	14	268	28	2	11	227	84	58
Fusobacteria				45			7	1
Gemmatimonadetes	11	1	2	2	9		7	40
Lentisphaerae			1					
Nitrospirae	3			7				2
NKB19			1					
OD1	1			15				19
OP11				4				5
OP3				4				8
Planctomycetes	25	9	6	181	13	3	10	218
Proteobacteria	482	123	213	952	309	94	275	798
Spirocahetes							1	
SR1				1				1
Tenericutes		1	1					
Thermi	3		1	2			1	2
TM6				11				11
TM7	16	2	11	29	7	1	7	23
Verrucomicrobia	51	2	17	117	26		19	125
WPS-2	1			9		4	2	21

WS2				3				1
WS3				3				4
Sub-total		517	472	2,489		447	764	2,292
Total	1,109		3,478		678		3,503	

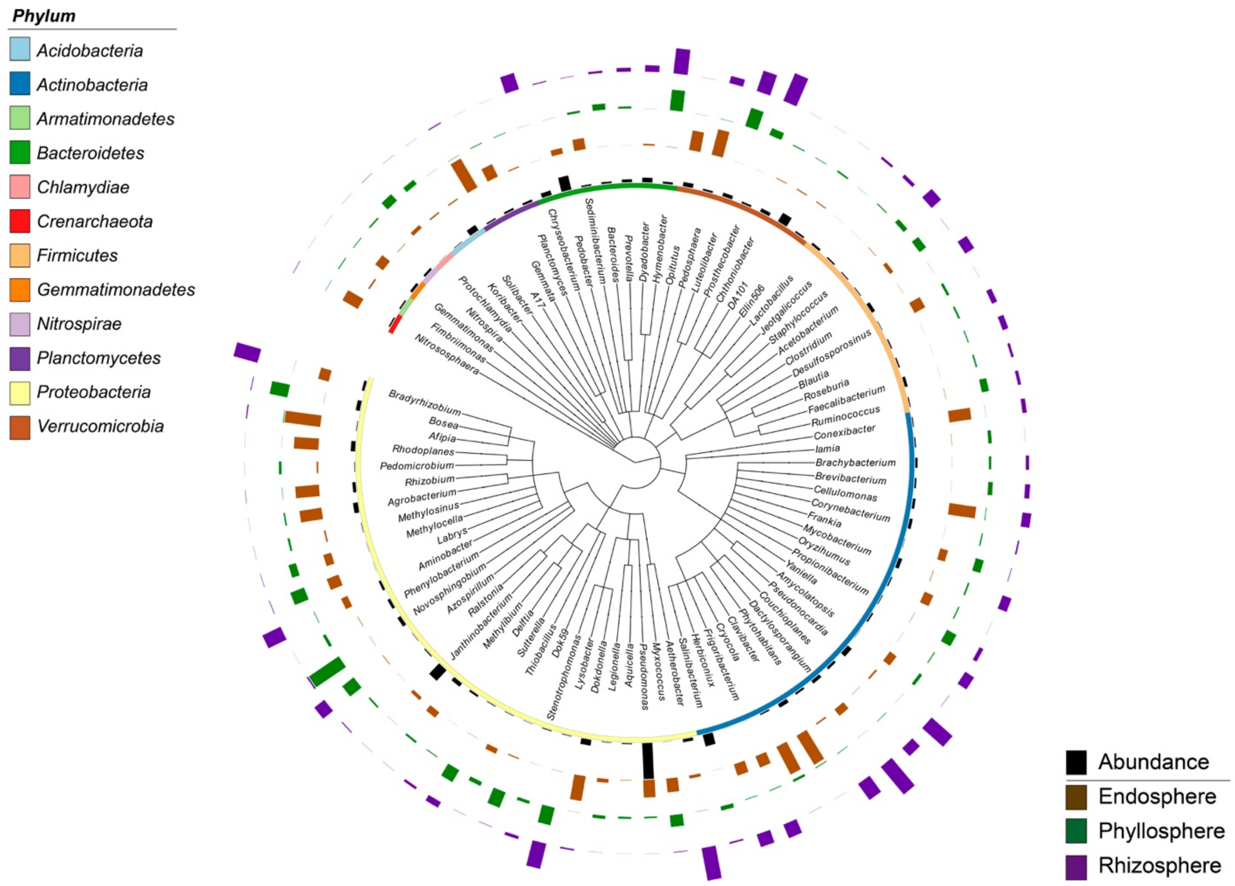

Figure S1. Indicator tree showing the taxonomy and taxon-treatment-association strength of 84 microbial genera significantly (q<0.1) associated with different compartments within Antarctic plants. Colors within the inner circle identify phyla. Heights of black bars in the innermost ring represent the square-root transformed abundances of the corresponding genera in the overall community. Heights of the colored bars on the remaining rings represent the relative, significant, association-strength (q<0.1) between the corresponding genera and the different compartments in Antarctic plants.
